# The emerging role of long non-coding RNAs, microRNAs, and an accelerated epigenetic age in Huntington’s disease

**DOI:** 10.3389/fnagi.2022.987174

**Published:** 2022-09-15

**Authors:** Soudeh Ghafouri-Fard, Tayyebeh Khoshbakht, Bashdar Mahmud Hussen, Mohammad Taheri, Kaveh Ebrahimzadeh, Rezvan Noroozi

**Affiliations:** ^1^Department of Medical Genetics, School of Medicine, Shahid Beheshti University of Medical Sciences, Tehran, Iran; ^2^Phytochemistry Research Center, Shahid Beheshti University of Medical Sciences, Tehran, Iran; ^3^Department of Pharmacognosy, College of Pharmacy, Hawler Medical University, Erbil, Iraq; ^4^Center of Research and Strategic Studies, Lebanese French University, Erbil, Iraq; ^5^Institute of Human Genetics, Jena University Hospital, Jena, Germany; ^6^Urology and Nephrology Research Center, Shahid Beheshti University of Medical Sciences, Tehran, Iran; ^7^Skull Base Research Center, Loghman Hakim Hospital, Shahid Beheshti University of Medical Sciences, Tehran, Iran; ^8^Malopolska Centre of Biotechnology, Jagiellonian University, Krakow, Poland

**Keywords:** Huntington’s disease, miRNA, lncRNA, DNA methylation, epigenetic age

## Abstract

Huntington’s disease (HD) is a dominantly inherited neurodegenerative disease with variable clinical manifestations. Recent studies highlighted the contribution of epigenetic alterations to HD progress and onset. The potential crosstalk between different epigenetic layers and players such as aberrant expression of non-coding RNAs and methylation alterations has been found to affect the pathogenesis of HD or mediate the effects of trinucleotide expansion in its pathophysiology. Also, microRNAs have been assessed for their roles in the modulation of HD manifestations, among them are miR-124, miR-128a, hsa-miR-323b-3p, miR-432, miR-146a, miR-19a, miR-27a, miR-101, miR-9*, miR-22, miR-132, and miR-214. Moreover, long non-coding RNAs such as DNM3OS, NEAT1, Meg3, and Abhd11os are suggested to be involved in the pathogenesis of HD. An accelerated DNA methylation age is another epigenetic signature reported recently for HD. The current literature search collected recent findings of dysregulation of miRNAs or lncRNAs as well as methylation changes and epigenetic age in HD.

## Introduction

Huntington’s disease (HD) is a neurodegenerative condition that is inherited in a Mendelian dominant fashion. This disorder has a wide variation in the age of onset. The disease is usually manifested in the 40 s with uncontrolled choreiform movements, cognitive defects, mood disorder, and behavioral alterations. HD is classified as a trinucleotide repeat disorder, resulting from increased numbers of CAG repeats in the *Huntingtin* gene (Myers, 2004). This disorder has variable clinical manifestations in terms of age of onset and severity of movement and cognitive functions. More than 50% of the variability in age of onset of HD is attributed to the size of the CAG repeat (Tabrizi et al., 2009). Persons with longer repeats usually have an earlier onset (Tabrizi et al., 2009). Nevertheless, subclinical alterations occur before the initiation of evident clinical manifestations. These changes include alterations in cognitive functions (Paulsen et al., 2006) as well as motor and oculomotor assessments (Gordon et al., 2000; Kirkwood et al., 2000).

Non-coding RNAs have been found to affect the pathobiology of HD or mediate the effects of trinucleotide expansion in its pathophysiology (Dubois et al., 2021; Tan et al., 2021). Particularly, two classes of non-coding RNAs, namely microRNAs (miRNAs) and long non-coding RNAs (lncRNAs) have been verified to be abnormally expressed in HD (Johnson, 2012; Dong and Cong, 2021b; Tan et al., 2021). These two classes of non-coding RNAs have been classified based on their size using the cutoff value of 200 nt. There are approximately 50,000 lncRNAs in the human genome (Cabili et al., 2011; Iyer et al., 2015). These transcripts partake in epigenetic mechanisms that influence chromatin configuration. Moreover, they are involved in the regulation of mRNA stability and imprinting processes (Managadze et al., 2013). LncRNAs are classified into five distinct groups, namely long intergenic non-coding RNAs, bidirectional, intronic, sense, and antisense lncRNAs (Ma et al., 2013). miRNAs are a distinct group of non-coding RNAs with sizes of about 22 nucleotides, regulatory roles in the expression of genes, and a high level of conservation among species (Bushati and Cohen, 2007). Both classes of non-coding RNAs are expressed in the brain and have important functions in the pathophysiology of neurodegenerative disorders (Sattari et al., 2020; Zhou et al., 2021).

Also, several studies explored different aspects of methylation alterations related to HD status (Vashishtha et al., 2013; Villar-Menéndez et al., 2013; Valor and Guiretti, 2014; Glajch and Sadri-Vakili, 2015; De Souza et al., 2016). Investigating the methylation signature of HD revealed the impact of specific methylation changes on disease progression and onset which can be caused by mutation or act through altering gene expressions (Vashishtha et al., 2013; Villar-Menéndez et al., 2013; Valor and Guiretti, 2014; Glajch and Sadri-Vakili, 2015; De Souza et al., 2016). DNA methylation estimated biological age (DNAm Age) of HD brain is reported to be accelerated in affected or non-affected regions. But the mechanisms underlying these methylation alterations are unclear which might be through a lncRNA-dependent manner (Vashishtha et al., 2013; Villar-Menéndez et al., 2013; Valor and Guiretti, 2014; Glajch and Sadri-Vakili, 2015; De Souza et al., 2016).

We designed the current study to collect information about the dysregulation of miRNAs and lncRNAs as well as DNA methylation changes in HD.

## MicroRNAs in Huntington’s disease

RNA-sequencing has enabled researchers to quantify miRNA expression in different brain regions (Langfelder et al., 2018). A similar experiment in the mice model of HD has revealed CAG length-dependent alterations in miRNA expression profile in the brain. Notably, selective alterations in expression profiles have been identified in 159, 102, 51, and 45 miRNAs in the striatum, cerebellum, hippocampus, and cortex, respectively (Langfelder et al., 2018).

miR-124 is among dysregulated miRNAs in HD. This miRNA has an important role in neurogenesis by regulating a few target genes (Cheng et al., 2009). Expression of miR-124 is decreased in the brain tissue of the HDR6/2 mice, expressing mHTT as well as in affected human subjects (Das et al., 2013). Expression of this miRNA has also been shown to be reduced in the cell and animal models of HD which express mutant HTT. Consistently, both models have exhibited up-regulation of levels of CCNA2, a predicted target of miR-124 (Das et al., 2013). Cumulatively, down-regulation of miR-124 can result in enhancement of expression of CCNA2 in these models of HD and subsequent deregulation of the cell cycle in affected cells (Das et al., 2013).

**FIGURE 1:**
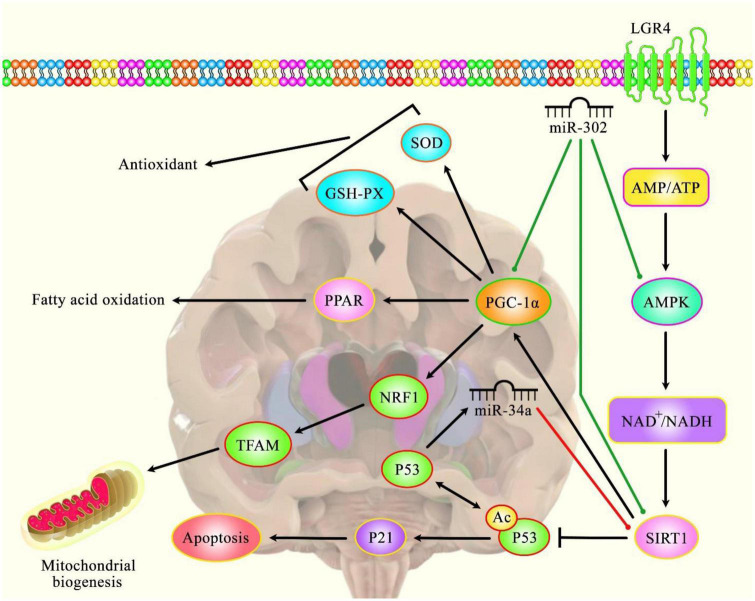
A schematic diagram of the role of several miRNAs in the modulation of Huntington’s disease.

Experiments in HD transgenic mice (R6/2 HD mice) have shown that miR-124 injection improves behavioral phenotype as evident by an increase in the latency to fall in the rotarod test (Liu et al., 2015). Furthermore, injection of this miRNA into bilateral striata has resulted in up-regulation of the neuroprotective factors PGC-1α and BDNF (Liu et al., 2015). Moreover, it has led to down-regulation of the repressor of cell differentiation SOX9 (Liu et al., 2015). Taken together, miR-124 can slow down HD course most probably *via* its vital functions in the differentiation and survival of neurons (Liu et al., 2015). Based on the important role of this miRNA in neurogenesis, Lee et al. have established an exosome-based delivery system to up-regulate miR-124 levels. Injection of Exo-124 into the striatum of R6/2 HD animal models has resulted in the reduction of expression of its target gene, REST. Yet, this strategy has not improved HD-related behavioral changes (Lee et al., 2017). Figure 1 illustrates the role of several miRNAs in regulating HD.

Accumulating evidence has illustrated that various miRNAs are an important regulatory factor in the pathoetiology of HD. It has been reported that miR-302 could play a crucial role in attenuating mHtt-induced cytotoxicity by promoting insulin sensitivity, resulting in a diminution of mHtt aggregates *via* the improvement of autophagy (Chang et al., 2021). Furthermore, miR-302 could also enhance the expression levels of silent information regulator 1 (Sirt1), AMP-activated protein kinase (AMPK), and PPARγ coactivator-1α (PGC-1α), therefore preserving mitochondrial function (Chang et al., 2021). The effect of the AMPK-SIRT1-PGC1α signaling pathway on antagonizing oxidative stress and maintaining mitochondrial function revealed an enhanced cognitive function in Sevo-anesthetized aged rats (Yang et al., 2020). SIRT1 as an NAD+ -dependent deacetylase has been investigated for its regulatory role in the regulation of cellular senescence and aging (Chen et al., 2020) and its association with lifespan elongation suggested it as a longevity gene (Bonkowski and Sinclair, 2016; Elibol and Kilic, 2018; Kim D. H. et al., 2019). Moreover, another research has figured out that dysregulation of p53/miR-34a/SIRT1 cascade could have an important role in HD-associated pathogenic mechanisms. Downregulation of miR-34a-5p could lead to the upregulation of SIRT1 and p53 protein levels in brain tissue (Reynolds et al., 2018). Green lines indicate the positive regulatory effect among miRNAs and their targets, and red lines depict the negative one among them. All the information regarding the role of these miRNAs participating in the pathobiology of Huntington’s disease can be seen in Table 1.

**TABLE 1:** MicroRNAs and Huntington’s disease (HD, Huntington’s disease; UHDRS, Unified Huntington’s Disease Rating Scales).

miRNA	Pattern of expression	Samples/ Animals	Cell lines	Targets/ Regulators/ Signaling pathways	Function	References
miR-124	decreased	R6/2 HD mice and their wild-type littermates	–	–	miR-124 reduces the progression of HD *via* promoting neurogenesis in the striatum.	Liu et al., 2015
	–	R6/2 HD mice	HEK 293 cells	REST	Overexpression of miR-124 reduced REST levels. But Exo-124 treatment did not lead to significant behavioral improvement.	Lee et al., 2017
	Decreased	R6/2 HD mice	STHdh(Q111)/Hdh(Q111) and STHdh(Q7)/Hdh(Q7) cells	CCNA2	Low levels of miR-124 could lead to high levels of CCNA2 in the cell and animal model of HD, thus it participates in the deregulation of the cell cycle.	Das et al., 2013
miR-128a	decreased	control, pre-symptomatic HD, and post-symptomatic HD human striatum samples/HD monkeys	–	HTT, HIP1	miR-128a has a role in regulation of HTT and HIP1.	Kocerha et al., 2014
hsa-miR-323b-3p	Increased	33 HD patients and 49 matched controls	–	HTT	A significant overconnectivity has been found between hsa-miR-323b-3p and HTT.	Ferraldeschi et al., 2021
miR-432 miR-146a miR-146a and miR-19a	Decreased	–	STHdh(Q111)/Hdh(Q111) and control STHdhQ7/HdhQ7cells	PCNA CHEK1 CCNA2	High expressions of these miRNAs in STHdh(Q111)/Hdh(Q111) cells relieved the irregularities in the cell cycle and apoptosis.	Das et al., 2015
miR-27a	Decreased	R6/2 HD mice	Primary neurosphere cells from C57BL/6 mice	mHtt, MDR-1	miR-27a could decrease mHtt levels of the HD cell by increasing MDR-1 function, thus playing a role in the reduction of mHtt aggregation in HD cells.	Ban et al., 2017
miR-34a	Decreased	R6/2 HD mice	–	SIRT1, p53	miR-34a was down-regulated and SIRT1 and p53 were up-regulated in HD, but, there were no known interactions between these factors.	Reynolds et al., 2018
miR-101	–	–	HEK293 cells	Rhes	miR-101 was found to target Rhes which plays an important role in HD development caused by striatal anomalies.	Mizuno and Taketomi, 2018
miR-9*	Decreased	36 HD patients and 28 healthy controls	–	–	miR-9* levels in peripheral leukocyte may be an indicator neurodegeneration in HD patients.	Chang et al., 2017
miR-22	Decreased	–	Primary cortical and striatal neuron cultures from striata or cerebral cortices of E16 rat embryos	HDAC4, Rcor1, and Rgs2	miR-22 has multipartite anti-neurodegenerative activities such as the inhibition of apoptosis *via* targeting HDAC4, Rcor1, and Rgs2.	Jovicic et al., 2013
miR-214	Increased	–	HD cell models	MFN2	miR-214 could increase the distribution of fragmented mitochondria and change the distribution of cells in different phases of the cell cycle by targeting MFN2.	Bucha et al., 2015
	Increased	–	Q7 and Q111 cells	Beta-catenin	Gain-of-function of mutant Htt could reduce beta-catenin levels *via* upregulating miR-214.	Ghatak and Raha, 2018
	Increased	_	STHdhQ7/Q7 and STHdhQ111/Q111 cells	Beta-catenin	miR-214 could reduce Beta-catenin post-transcriptionally, thus transcriptional activity of wnt/β-catenin signaling was decreased.	Ghatak and Raha, 2015
miR-196a	Decreased	R6/2 HD mice	N2a mouse neuroblastoma cells and primary neurons	RANBP10	miR-196a could increase neuronal morphology to provide neuroprotection in HD *via* targeting RANBP10.	Her et al., 2017
	Decreased	Analysis of different bioinformatics tools, including DAVID, MSigDB, TargetScan, and MetaCore	–	–	miR-196a could have beneficial functions *via* the alteration of cytoskeleton structures.	Fu et al., 2015
	Increased	Eight HD patients and four controls	WT-NPCs, HD-NPCs, and HD-NCs	–	miR-196a could reduce cytotoxicity and apoptosis in HD-NHP neural progenitor cells and differentiated neural cells.	Kunkanjanawan et al., 2016
	Decreased	D-Tg mice, GHD mice, 196a transgenic mice, and WT mice	293 FT cells, N2a cells, and HD-iPSCs	HTT	miR-196a could reduce mHTT in the brain and also improve neuropathological progression.	Cheng et al., 2013
hsa-miR-4324 and hsa-miR-4756-5p	–	HD patients	HEK293T cells and derived fibroblast from HD patients	–	hsa-miR-4324 and hsa-miR-4756-5p could reduce HTT 3’-UTR reporter activity and endogenous HTT protein levels.	Kim K. H. et al., 2019
miR-302	Decreased	–	SK-N-MC neuroblastoma cells	Sirt1/AMPK-PGC1α pathway	miR-302 could reduce mHtt-induced cytotoxicity by increasing insulin sensitivity, leading to a reduction of mHtt aggregates *via* the increasing autophagy, besides it could upregulate Sirt1/AMPK-PGC1α pathway.	Chang et al., 2021
miR-10b-5p	Increased	12 HD patients and nine control samples	–	BDNF	miR-10b-5p could reduce BDNF expression which is associated with neuronal dysfunction and death.	Müller, 2014
miR-10b-5p	Increased	prefrontal cortex samples of 26 HD patients and 36 controls	–	–	miR-10b-5p expression in brain tissues is correlated with to age of onset and the severity of striatal pathology.	Hoss et al., 2015

Another experiment in a monkey model of HD has shown dysregulation of 11 miRNAs in the frontal cortex of these animals among them being the down-regulated miRNA miR-128a. This miRNA has also been found to be down-regulated in the brain tissues obtained from both pre-symptomatic and post-symptomatic affected persons (Kocerha et al., 2014).

A study on the human subject has shown down-regulation of hsa-miR-98 and over-expression of hsa-miR-323b-3p in HD cases compared with healthy subjects and psychiatric patients (Ferraldeschi et al., 2021). Additional expression assays in an independent cohort of HD cases have validated up-regulation of hsa-miR-323b-3p in HD cases even before disease manifestations (Ferraldeschi et al., 2021). However, authors have reported no significant difference in expression of hsa-miR-98 in the second cohort. Further bioinformatics evaluations have shown overconnectivity between the hsa-miR-323b-3p targetome and the HTT interactome. Besides, these investigations have shown transcriptional regulation of the HTT interactome by the targetome of this miRNA (Ferraldeschi et al., 2021).

Experiments in a cell model of HD have confirmed the delay in the S and G2-M phases of cell cycles in these cells compared to control cells (Das et al., 2015). Consistent with this finding, expressions of PCNA, CHEK1, and CCNA2 are elevated in primary cortical neurons expressing mutant Huntingtin (mHTT) as well as animal and cell models of HD. Over-expression of these genes has resulted from down-regulation of miR-432, miR-146a, and miR-19a, and miR-146a, respectively (Das et al., 2015).

miR-27a is another down-regulated miRNA in the brain tissues of the HD mice model. This miRNA can regulate the expression of MDR-1 (Ban et al., 2017). Transfection of miR-27a into the differentiated neuronal stem cells originating from the R6/2 HD mouse model has led to decreased mHtt aggregation. Furthermore, the level of MDR-1, as a transporter of mHtt, has been enhanced by this miRNA. Thus, miR-27a can decrease mHtt levels in the HD cells through increasing MDR-1 efflux function (Ban et al., 2017).

**FIGURE 2:**
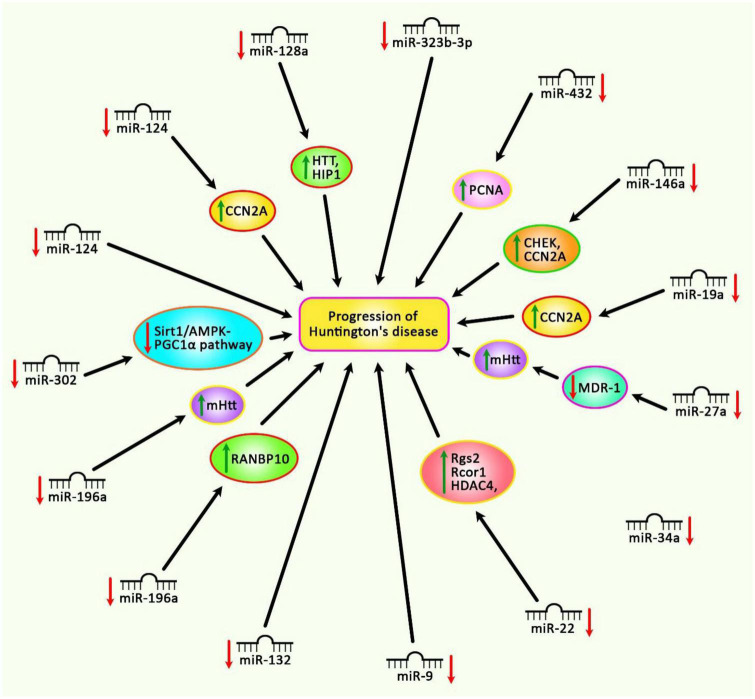
Different experiments have shown the down-regulation of miRNAs in Huntington’s disease. Subsequent up-regulation of mRNA targets of these miRNAs can lead to the progression of Huntington’s disease.

Figure 2 summarizes the impact of down-regulated miRNAs in the pathogenesis of HD.

On the other hand, a quantity of miRNAs has been reported to be up-regulated in HD. For instance, miR-214 has been found to be increased in HD cell model (Bucha et al., 2015). This miRNA can target HTT mRNA. Moreover, expression levels of numerous HTT co-expressed genes have been demonstrated to be affected by exogenous expression of mutant or wildtype miR-214. MFN2 is an example of HTT co-expressed genes which is directly targeted by miR-214 (Bucha et al., 2015). Over-expression of miR-214 could result in repression of MFN2 expression, increase in the dispersal of fragmented mitochondria and changes in the distribution of cells in diverse stages of the cell cycle. Taken together, up-regulation of miR-214 can affect the morphology of mitochondria and disturb cell cycle regulation in HD models (Bucha et al., 2015). Another study has shown up-regulation of miR-214 following gain-of-function mutation in Htt in Q7 and Q111 HD cells. This miRNA could also decrease β-catenin levels and its transcriptional activity (Ghatak and Raha, 2018).

**FIGURE 3:**
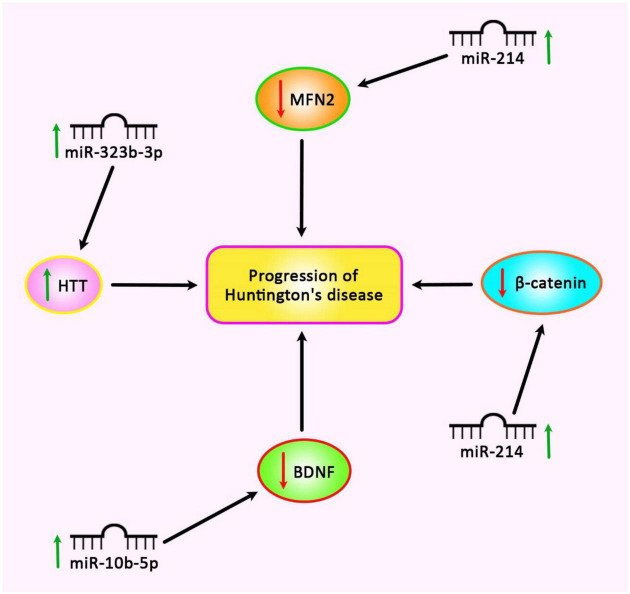
Up-regulation of miR-214, miR-322b-3p, and miR-10b-5p is involved in the pathogenesis of Huntington’s disease.

Re-analysis of the high throughput expression data has shown dysregulation of several miRNAs in HD. Further studies have shown that up-regulated miRNAs, miR-10b-5p, and miR-30a-5p can regulate the expression of BDNF (Müller, 2014). Down-regulation of BDNF is correlated with dysfunction and death of neurons in HD (Müller, 2014). Besides, these two miRNAs have been predicted to target CREB1, a down-regulated gene in HD whose up-regulation can decrease susceptibility to 3-NP-induced toxicity (Chaturvedi et al., 2012). Contradictory to these results, it is supposed that up-regulation of miR-10b-5p plays a neuroprotective role against *HTT* mutation (Müller, 2014). Thus, the functional role of miR-10b-5p and its impact on the expression of BDNF in HD need additional investigations (Müller, 2014). Figure 3 shows up-regulated miRNAs in HD.

Soldati et al. (2013) have assessed the impact of up-regulation of nuclear REST on the expression of miRNAs in the presence of mHTT. Comparison of expression levels of 41 miRNAs in *Hdh*^109/109^ cells and *Hdh*^7/7^ cells has led to the identification of 15 down-regulation miRNAs in the former group, including miR-9, miR-9*, and miR-23b. The expression of 12 miRNAs among these down-regulated miRNAs (including the three mentioned miRNAs) have been enhanced after REST knock-down. Notably, miR-137, miR-153, and miR-455 are mouse homologs of human miRNAs with predicted REST binding sites. Authors have concluded that several miRNAs with abnormal expression in HD are presumably suppressed by over-expressed REST (Soldati et al., 2013).

Analysis of expression of miRNAs in the postmortem brain samples of HD patients has resulted in the identification of differential expression of 54 miRNAs, including 30 upregulated and 24 downregulated miRNAs. Expressions of 26 miRNAs have been found by several number of transcription factors, namely TP53, E2F1, REST, and GATA4 (Sinha et al., 2012).

In fact, repression of expression of important neuronal miRNAs such as mir-9/9*, mir-124, and mir-132-is in the brain regions of HD patients and animal models occurs downstream of REST, possibly due to interruption of mRNA regulation and neuron functions. In this study, we will discuss these findings and their implications for our understanding of HD. An *in silico* assessment has led to the prediction of 21 novel candidate miRNAs in HD. This study has indicated that HD is associated with a large-scale suppression of neural genes in the caudate and motor cortex. Moreover, it has been concluded that cooperation between REST, miRNAs and probably other non-coding RNAs can significantly affect the transcriptome of neurons in HD (Johnson and Buckley, 2009). Mechanistically, polyglutamine expansion in huntingtin has been shown to abrogate REST-huntingtin binding. This leads to nuclear translocation of REST (Zuccato et al., 2003) where it lodges RE1 repressor sequences and reduces gene expression in neurons (Zuccato et al., 2003). Packer et al. (2008) have reported the reduction of expression of numerous miRNAs with upstream RE1 sites in cortex samples of HD patients compared with healthy controls. Notably, among these miRNAs has been the bifunctional brain enriched miR-9/miR-9* which targets two constituents of the REST complex (Packer et al., 2008).

Another experiment in post-mortem tissues of HD patients has demonstrated accumulation of Argonaute-2 (AGO2) in the presence of neuronal protein aggregates as a result of impairment of autophagy. Since AGO2 is an important constituent of the RISC complex that implements miRNA functions, its accumulation leads to global changes in the miRNA levels and activity (Pircs et al., 2018).

Finally, experiments in the 3NP-induced animal model of HD have shown distinct miRNA profiles compared with the transgenic mice. This observation is possibly due to the effects of mHtt on the activity of HTT in extra-mitochondrial energy metabolism (Lee et al., 2007).

Table 1 lists miRNAs that are possibly involved in the pathogenesis of HD.

Most notably, investigations in animal models of HD have demonstrated that artificial miRNAs are able to reduce levels of mHTT. Pfister et al. (2018) have performed an experiment in HD transgenic sheep model that expresses the full-length human HTT with 73 CAG repeats. Treatment of these animals with AAV9-expressing an artificial miRNA targeting exon 48 of the human HTT transcript has led to a reduction of human mHTT transcript and protein in the striatum without any significant neuron loss. This study has revealed the safety and efficiency of silencing human mHTT protein using an AAV-mediated transfer of an artificial miRNA (Pfister et al., 2018).

## Long non-coding RNAs in Huntington’s disease

Few lncRNAs are dysregulated in HD (Table 2). Expression of lncRNA-DNM3OS has been assessed in a rat pheochromocytoma cell line induced by *Huntingtin* gene exon 1 fragment containing either 23 or 74 CAG repeats. This intervention has led to up-regulation of GAPDH and DNM3OS. Down-regulation of these genes has resulted in suppression of aggregate formation, reduction of apoptosis and enhancement of cell survival. Furthermore, up-regulation of DNM3OS in HD PC12 cells can decrease miR-196b-5p levels by sponging. GAPDH is a direct target of this miRNA which contributes in the development of aggregates (Dong and Cong, 2021a).

**TABLE 2:** Long non-coding RNAs and Huntington’s disease (HD, Huntington’s disease; mHTT, mutant Huntingtin).

lncRNA	Pattern of expression	Samples/ Animals	Cell lines	Targets/ Regulators/ Signaling pathways	Description	References
DNM3OS	increased	–	HD PC12 cells (httex1p−Q23 and httex1p−Q74)	miR-196b-5p/GAPDH	Downregulation of DNM3OS leads to suppression of aggregate formation accompanied by a reduced apoptosis and augmented relative ROS levels and cell viability.	Dong and Cong, 2021a
NEAT1	Increased	R6/2 HD mice	neuro2A cells	–	Upregulation of NEAT1 could increase viability under oxidative stress.	Sunwoo et al., 2017
	Increased	HD mice	STHdhQ7/Q7 cells and STHdhQ111/Q111	mHTT, MeCP2	The elevation of NEAT1 was mHTT dependent, as knockdown of mHTT restored Neat1L to normal levels. It was found that Neat1L is suppressed by MeCP2 *via* RNA-protein interaction.	Cheng et al., 2018
Meg3 and NEAT1	Increased	R6/2 HD mice	STHdhQ7/HdhQ7 cells and STHdhQ111/HdhQ111 cells	–	Downregulation of Meg3 and NEAT1 could decrease aggregate formation by mHTT and downregulation of Tp53 expression.	Chanda et al., 2018
Abhd11os	Decreased	male C57BL/6J mice	HEK293T cells	–	Upregulation of Abhd11os protects neurons against an N-terminal fragment of mHTT, while Abhd11os downregulation is protoxic.	Francelle et al., 2015

**FIGURE 4:**
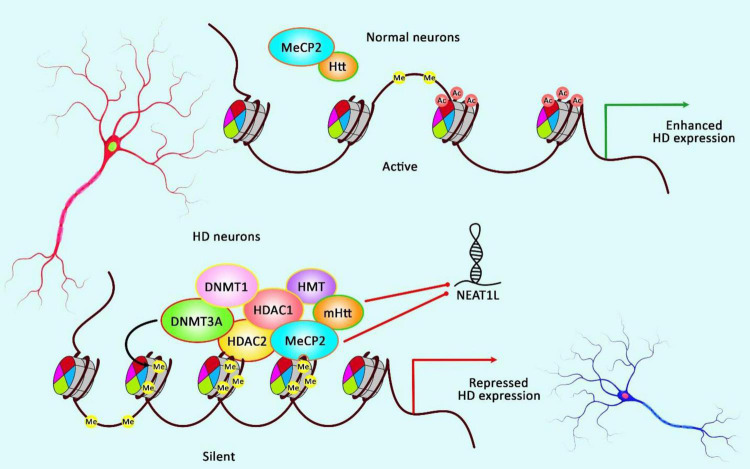
A schematic illustration of the role of NEAT1L in HD.

Sunwoo et al. (2017) have performed a microarray-based study to evaluate the expression profile of lncRNAs in HD. They have reported up-regulation of NEAT1 (Sunwoo et al., 2017). Further studies in brain tissues of R6/2 HD models as well as postmortem brain tissues of human HD subjects have validated the up-regulation of this lncRNA (Sunwoo et al., 2017). Functional studies have shown increased viability of cells in oxidative stress conditions following transfection of cells with NEAT1. Taken together, up-regulation of NEAT1 in HD can be regarded as a neuroprotective strategy against neuronal damage instead of being a pathological event (Sunwoo et al., 2017). Another study has revealed up-regulation of the long isoform of NEAT1 in the brain tissues of mice model of HD. This transcript has also been up-regulated in differentiated striatal neurons originating from HD knock-in mice as well as brain tissues of affected individuals. Up-regulation of this isoform has been found to be dependent on mHTT. MeCP2 could repress expression of this isoform of Neat1 through a molecular interaction outside its promoter. Up-regulation of this isoform exerts a protective effect against mHTT-induced cytotoxicity. On the other hand, its down-regulation results in impairment of several cellular processes such as those related with cell proliferation and development. Interestingly, dysregulated genes in HD human brain tissues overlap with pathways influenced by down-regulation of NEAT1 (Figure 4; Cheng et al., 2018).

Mounting evidence has demonstrated that aberrant expression of various lncRNAs could be correlated with dysfunction and death of neurons in HD (Cheng et al., 2018). As an illustration, a recent study has detected that the expression of NEAT1L could be inhibited by mHTT as well as MeCP2 *via* RNA-protein interaction, thereby NEAT1L may play a protective role in CAG-repeat expansion diseases, such as HD (Cheng et al., 2018).

Chanda et al. (2018) have used small RNA sequencing and PCR array techniques to find dysregulated RNAs in R6/2 HD mice brains. They have detected alteration in 12 non-coding RNAs in these samples eight of them having human homologs. Three lncRNAs, namely Meg3, Neat1, and Xist have exhibited a constant and substantial over-expression in cell and animal models of HD. Silencing of Meg3 and Neat1 in cell models of HD has resulted in the reduction of aggregate formation by mHTT and a significant decrease in the endogenous levels of Tp53 (Chanda et al., 2018).

**FIGURE 5:**
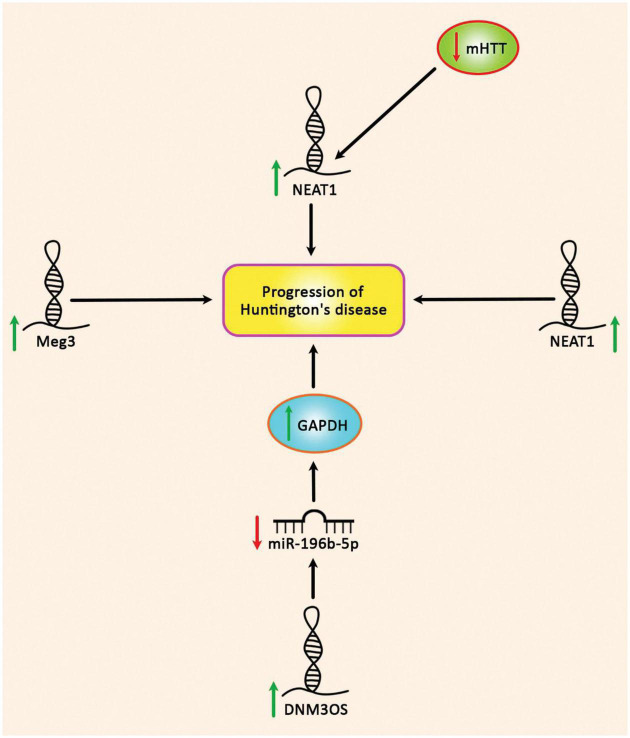
Up-regulation of DNM3OS, NEAT1, and Meg3 in Huntington’s disease.

Abhd11os is another lncRNA that is down-regulated in various animal models of HD. Up-regulation of Abhd11os has a neuroprotective effect against an N-terminal fragment of mHTT, while its silencing has a toxic effect. Thus, loss of Abhd11os contributes to striatal susceptibility in HD (Francelle et al., 2015). Figure 5 depicts up-regulated lncRNAs in HD.

## DNA methylation age acceleration in Huntington’s disease

The crosstalk among different epigenetic players such as histone modification, non-coding RNA action, and DNA methylation, ensures stable regulation of gene expression required for the maintenance of cell-type-specific identity. However, environmentally induced alterations in the epigenome may lead to chromatin remodeling, and as a result, the abnormal gene expression can affect the state of differentiated cells (Boland et al., 2014). In the case of neuronal cells, the interplay between epigenetic mechanisms and transcriptional changes controls neuronal cell identity as well, but it also regulates neuronal activation and consequent brain functions such as cognitive and motor functions in response to environmental signals (Francelle et al., 2017).

One of the main epigenetic mechanisms that have profound effects on the DNA packaging, gene expression, and maintenance of the cellular identity, is DNA methylation, the addition of a methyl group (CH3), particularly to the fifth position of carbon in the cytosine ring, which results in the formation of 5-methylcytosine (5mC), and creates a mutational hotspot in the genome which may finally affect the susceptibility to different diseases (Denissenko et al., 1997).

Recently, the DNA methylation levels of CpG markers, correlated with age or age-related physiological dysregulation, have been used to develop different DNA methylation-based age estimators, also known as DNA methylation biological age clocks (Horvath, 2013). Tissue-specific DNA methylation variability has been correlated with the differences in biological ages of individuals and the estimated biological age is reported to be accelerated in different diseases including cognitive and neurodegenerative disorders (Noroozi et al., 2021). The aging-related systematic decline and biological aging have been shown to play an active role in HD pathogenesis, its onset, and progression (Machiela and Southwell, 2020). And recent research studies aim to reveal the role of methylation modifications in HD pathogenesis (Lee et al., 2013; Valor and Guiretti, 2014). The unstable CAG trinucleotide expansion in HD is suggested to be affected by the biological age and the methylation pattern of the tissue. The transcriptional dysregulation observed in the HD brain tissue is reported to be influenced by aberrant DNA methylation (De Souza et al., 2016) which modulates the expression levels of *HTT* and other neuronal identity genes (Hyeon et al., 2021). DNA methylation alterations in response to polyglutamine-expanded *HTT* were observed in HD cell models at both promoter-proximal and distal regulatory regions (Ng et al., 2013). A tissue-specific methylation pattern of the *HTT* gene was reported by analyzing post-mortem cortex and liver tissues of HD patients (De Souza et al., 2016).

Investigation of the DNA methylation changes in the post-mortem brain tissues of HD patients demonstrates accelerated epigenetic aging (Horvath et al., 2016). The recent epigenome-wide association study (EWAS) of HD gene-expansion carriers (HDGECs) in multiple tissues from three species, reported 33 genome-wide significant CpGs for HD status and the most significant locus was *HTT* and the EWAS results of motor progression in manifest HD cases indicate significant associations with methylation levels at three loci. Also, a higher blood epigenetic age acceleration (calculated discrepancy between chronological age and DNA methylation age) was reported in manifest HD in comparison to controls (Lu et al., 2020). Most recently, exploration of the HD-associated epigenetic signatures using the slowly progressing knockin (KI) mouse model of HD, revealed that even before the onset of HD motor problems, age-related epigenetic remodeling and transcriptional alteration of neuronal and glial-specific genes are accelerated. This might suggest that the progressive HD striatal epigenetic signatures develop from the earliest stages of HD (Alcalá-Vida et al., 2021).

## Discussion

Neurodegenerative processes in the brains of HD patients are associated with extensive alterations in gene regulatory networks. These alterations have been detected in both protein-coding genes and non-coding RNAs (ncRNAs) (Johnson, 2012). Among non-coding RNAs, the impacts of miRNAs in HD pathogenesis have been investigated more than lncRNAs. LncRNAs can inhibit or stimulate the neurodegenerative processes through epigenetically modulating expression of genes critically implicated in the pathoetiology of these disorders (Zhou et al., 2021). Thus, further high throughput studies are needed to find other HD-related lncRNAs.

The functional effects of dysregulation of non-coding RNAs in the development of HD have been assessed in cell and animal models of HD. These transcripts mostly affect the survival of neurons through influencing apoptotic pathways or cell cycle transition.

The expression profile of non-coding RNAs in the circulation of suspected persons might be used to predict HD course. Thus, these transcripts can be used as biomarkers for HD. They can also be targeted by siRNAs or antisense oligonucleotides. Therefore, they represent potential targets for gene therapy of HD. It is worth mentioning that altered expression of non-coding RNAs in HD might be either a part of pathogenic processes in this disorder or a compensatory mechanism for increasing the viability of neurons. This note should be considered in the design of therapeutic modalities.

In addition, the pathophysiology of HD is affected by epigenetic modulations and the methylation levels of selected genes are related to HD progress and onset. Accelerated epigenetic age of HD brain suggested association of the older biological age of the affected tissue with the disease which is not affected by sex, age, or the abundance of neuronal cells. Enrichment analysis on the methylation modules of HD highlights the association of genes involved in olfactory receptor activity and sensory perception of chemical stimulus with HD status.

The crosstalk between DNAm and non-coding RNAs, and most interestingly regulation of DNA methylation by lncRNAs is an important factor that can declare the underlying mechanisms of neurodegenerative disorders. The association analysis of the methylation levels of different age-related loci in blood and buccal swab samples showed a significant association between the hypomethylation of a lncRNA MIR29B2CHG with age, suggesting it is a marker for the development of DNAm based age prediction models in these tissues (Tharakan et al., 2020; Woźniak et al., 2021).

Several miRNAs have been shown to affect the expression of mHTT, thus they can be used as modulators of HD pathogenic events. Moreover, several panels of miRNAs are dysregulated in the course of HD, thus having the potential to be used as predictive markers in this disorder. Several number of dysregulated miRNAs in HD are regarded as specific markers for this disorder, thus being promising biomarkers for diagnostic purposes as well as monitoring HD progression and therapeutic response.

Besides, lncRNAs have been suggested to play an active role in specifying the pattern of histone modifications of target genes by serving as scaffolds for histone modification enzymes. For example, the binding of LSD1 enzyme to target genes, mediated by *HOTAIR*, has been shown to affect the repression of target genes by changing the methylation level of the histone H3 lysine 27 (H3K27) (Tsai et al., 2010). While, decreased acetylation of H3K27 showed an association with the downregulation of neuronal identity genes in the striatum of HD patients and mice (Achour et al., 2015; Alcalá-Vida et al., 2021). Also, an association is shown between aberrant DNA methylation levels correlated with aberrant expression of different lncRNAs with susceptibility to different diseases. For instance, altered expression of *FMR4* lncRNA is reported to change genome-wide histone methylations and play an active role in the development of repeat expansion-associated disorders such as fragile X (Peschansky et al., 2016).

Although investigating the epigenetic signatures of the HD suggested these alterations to be the consequent rather than cause of the underlying genetic structure of the HD (Zimmer-Bensch, 2020), potential interactions between different layers of epigenome for instance the lncRNA-dependent methylation alterations related to HD progress and onset should be investigated with much rigor.

## Author contributions

SG-F wrote the manuscript and revised it. MT and KE supervised and designed the study. TK, RN, and BH collected the data and designed the figures and tables. All authors have read and approved the submitted version.
